# Discrimination of the Geographical Origin of Soybeans Using NMR-Based Metabolomics

**DOI:** 10.3390/foods10020435

**Published:** 2021-02-17

**Authors:** Yaoyao Zhou, Seok-Young Kim, Jae-Soung Lee, Byeung-Kon Shin, Jeong-Ah Seo, Young-Suk Kim, Do-Yup Lee, Hyung-Kyoon Choi

**Affiliations:** 1 College of Pharmacy, Chung-Ang University, Seoul 06974, Korea; zhouyaoyao.zy@gmail.com (Y.Z.); dud612@naver.com (S.-Y.K.); iverson1989@naver.com (J.-S.L.); 2 National Agricultural Products Quality Management Service, Gimcheon 39660, Korea; sbkon1@korea.kr; 3 School of Systems Biomedical Science, Soongsil University, Seoul 06978, Korea; sja815@ssu.ac.kr; 4 Department of Food Science and Engineering, Ewha Womans University, Seoul 03760, Korea; yskim10@ewha.ac.kr; 5 Center for Food and Bioconvergence, Department of Agricultural Biotechnology, Research Institute for Agricultural and Life Sciences, CALS, Seoul National University, Seoul 08826, Korea

**Keywords:** metabolic profiling, *Glycine max*, NMR, geographical location, prediction

## Abstract

With the increase in soybean trade between countries, the intentional mislabeling of the origin of soybeans has become a serious problem worldwide. In this study, metabolic profiling of soybeans from the Republic of Korea and China was performed by nuclear magnetic resonance (NMR) spectroscopy coupled with multivariate statistical analysis to predict the geographical origin of soybeans. The optimal orthogonal partial least squares-discriminant analysis (OPLS-DA) model was obtained using total area normalization and unit variance (UV) scaling, without applying the variable influences on projection (VIP) cut-off value, resulting in 96.9% sensitivity, 94.4% specificity, and 95.6% accuracy in the leave-one-out cross validation (LOO-CV) test for discriminating between Korean and Chinese soybeans. Soybeans from the northeastern, middle, and southern regions of China were successfully differentiated by standardized area normalization and UV scaling with a VIP cut-off value of 1.0, resulting in 100% sensitivity, 91.7%–100% specificity, and 94.4%–100% accuracy in a LOO-CV test. The methods employed in this study can be used to obtain essential information for the authentication of soybean samples from diverse geographical locations in future studies.

## 1. Introduction

Soybean (*Glycine max* L. Merr.) is an important legume for food, animal feed, and biofuels [1]. Soybean contains 40% protein; therefore, it is a common, rich and easily accessible protein source. It is also rich in soluble sugars/dietary fiber (35%) and oil (20%), 85% of which is cholesterol-free, and contains both monounsaturated and polyunsaturated fatty acids [2]. Soybean metabolites exhibit a wide range of pharmacologically beneficial effects, including antioxidant, hypo-cholesterolemic, anticarcinogenic, immunostimulatory, antidiabetic activities, and reduction of osteoporosis risk [2,3].

With the increase in international agricultural trade and consumer demand for safe and high-quality food, identifying the geographical origin of agricultural products has increased in importance. Soybeans are one of the most traded agricultural products worldwide. As soybean trade between countries increases, some soybean distributors and processing companies mix relatively inexpensive imported soybeans with domestic soybeans and sell them without revealing the exact source of origin information. Identifying the geographical origin of soybeans is important from several perspectives. First, the safety issues of genetically modified (GM) soybeans and low-quality imported soybeans should be considered. More than half of the soybeans produced worldwide are GM soybeans; this has raised consumer concerns regarding allergy risks [4,5]. In addition, there are quality degradation issues, such as microbial contamination and pesticide detection, during the storage and distribution of imported soybeans. Second, regarding the nutritional properties of soybeans, previous studies have shown that the levels of primary and secondary metabolites such as soybean sugars, proteins, fatty acids, and phytochemicals differ depending on the geographical origin [6,7]. Third, different soybean processing technologies are required depending on the soybean origin because the content of useful nutrients or anti-nutritional factors (trypsin inhibitors) varies. Processing technologies that can minimize the destruction of beneficial nutrients while removing unnecessary nutrients from the soybeans must be developed and applied appropriately depending on the origin [8]. Therefore, it is necessary to develop a method for determining the geographical origin of soybeans that can solve the problem of intentionally mislabeling the origin of soybeans, which has disrupted the soybean distribution system and created confusion among consumers.

Metabolomics has been widely used in previous studies to observe the changes in metabolites of agricultural products from different geographical origins using high-performance liquid chromatography (HPLC), gas chromatography (GC), capillary electrophoresis-time-of-flight (CE-TOF) combined with mass spectrometry (MS), nuclear magnetic resonance (NMR) spectroscopy, and Fourier-transform infrared (FT-IR) spectroscopy [9,10,11,12,13]. Among various analytical platforms, NMR spectroscopy has been widely used to characterize food resources because of its simultaneous detection of diverse compounds within a complex mixture, high reproducibility, and noninvasive nature [14,15]. Several studies have analyzed different soybean varieties using NMR, GC-MS, and ultra-performance liquid chromatography (UPLC)-tandem mass spectrometry (MS/MS) analyses [16,17]. The profiling of isoflavones and anthocyanins in black soybeans from different geographical locations in southwest China has been performed using HPLC-MS [7]. Metabolite fingerprinting has been widely used as an important tool for authentication of food and agricultural products [18]. This allows the determination of exact geographical origin and the detection of any unusual quality of raw materials in both unprocessed and processed products [18,19]. However, comparative and comprehensive metabolic profiling using NMR spectroscopy has rarely been applied to soybean samples from different geographical regions.

Accordingly, in the present study, soybean samples obtained from major soybean-producing areas and their local markets were used to establish a representative prediction model for discriminating the geographical origin of soybeans from Korea and China. NMR-based metabolic profiling coupled with multivariate statistical analysis was employed to determine the geographical origin of soybeans from Korea and China.

## 2. Materials and Methods

### 2.1. Soybean Samples

Authentic Korean soybeans from eight regions harvested in 2016 were provided from the National Agricultural Products Quality Management Service of the Republic of Korea. These were obtained from Gyeonggi-do Anseong, Gangwon-do Yeongwol, Chungcheongbuk-do Eumseong, Chungcheongnam-do Cheonan, Jeollabuk-do Imsil, Jeollanam-do Yeonggwang, Gyeongsangbuk-do Uiseong, and Gyeongsangnam-do Geochang, 500 g to 2 kg from each region. Chinese soybeans were purchased from online suppliers in October 2016. Nine Chinese samples were obtained from the three major and largest soybean producing regions including northeastern (Heilongjiang, Jilin, Liaoning), middle (Hebei, Shandong, and Hubei), and southern (Zhejiang, Guangdong, and Guangxi) regions, 500 g from each region, and the products and suppliers’ information of these soybean products are listed in Table S1. Geographical information on Korean and Chinese soybean samples is presented in Figure S1. Four replicates from each pool were collected for each region, ground in liquid nitrogen using a blender, freeze-dried, and then stored at −80 °C until NMR analysis. For measurement of size, 10 grains of soybean samples from each region were randomly selected and measured. The length along the hypocotyls was measured as soybean size using a digimatic caliper (0–150 mm, S. Tools, Wuxi, China).

### 2.2. Chemicals and Reagents

Methanol-d_4_ (MeOD, 99.8% atom D) and sodium deuteroxide (NaOD, 99.5% atom D; 40% in D_2_O) were obtained from Cambridge Isotope Laboratories, Inc. (Andover, MA, USA). Deuterium oxide (D_2_O, 99.9% atom D) including 0.05% 3-(trimethylsilyl) propionic-2, 2, 3, 3-d_4_ acid sodium salt (TSP) and potassium phosphate (KH_2_PO_4_) was purchased from Sigma-Aldrich (St. Louis, MO, USA).

### 2.3. Climate Data for Soybean Cultivation Regions in Korea and China

The monthly average temperature (°C) and total precipitation (mm) data for soybean cultivation regions in Korea and China in 2016 were obtained from the Korea Meteorological Administration (https://data.kma.go.kr) (accessed on 12 November 2020) and China Meteorological Administration (https://data.cma.cn/en) (accessed on 12 November 2020), respectively. The average annual temperature and precipitation were calculated by averaging monthly climate data for one year (January to December 2016).

### 2.4. NMR Measurement and Peak Assignment

To extract various groups of metabolites in soybeans, aqueous methanol (50% D_2_O-MeOD mixture) was used as an extraction solvent in this study [20,21]. Fifty milligrams of soybean powder and 1.5 mL of 50% D_2_O-methanol mixture (D_2_O was titrated to pH 6 using NaOD) were transferred into a 2-mL centrifuge tube, and then vortexed and sonicated for 1 min and 15 min, respectively. Thereafter, the material was centrifuged at 17,000× *g*, 4 °C for 10 min. Buffer solution of 0.1 M KH_2_PO_4_ was prepared from D_2_O and NaOD was used to adjust pH to 6. The clear supernatant was filtered with a 0.45-µm Whatman filter (PTFE, Sigma-Aldrich), and 600 µL of the sample was transferred into 5-mm NMR tube (Norell, Landisville, NJ, USA).

A 600-MHz Bruker Avance spectrometer (Bruker, Germany) was employed to analyze soybean samples at a temperature of ~25 °C to record all NMR spectra. For ^1^H-NMR spectra, 64 K data points were obtained with a relaxation delay of 2.0 s and a spectral width of 12626.3 Hz. A scan number of 128 and an acquisition time of 2.6 s were used. Water suppression was conducted to exclude the region between δ = 4.7 to 5.0. For two-dimensional NMR spectra, ^1^H-^1^H correlation spectroscopy (COSY) spectra were acquired under the following conditions: 32 scans, relaxation delay of 2.0 s, and 7812.5 Hz spectral width. ^1^H-^13^C heteronuclear single quantum correlation (HSQC) spectra were obtained with 32 scans, 2.0 s relaxation delay, and 6631.3 Hz spectral width. The following pulse sequence described by Suh et al. [22] was used to collect ^1^H-NMR spectra: relaxation delay –90°–t_1_–180°–t_1_–acquire for time t_2_. The relaxation delay of t_1_ was incremented together with the increasing delay, and the relaxation delay was 2.0 s for time t_2_. Baseline correction and assignments of all ^1^H-NMR spectra were performed by using Chenomx NMR suite software (version 8.2, Chenomx, Edmonton, AB, Canada) and further identification of metabolites was performed based on the HMDB database (HMDB, http://www.hmdb.ca/) (accessed on 18 January 2020). Non-overlapping peaks were used for the peak assignment. MestReNova (version 6.0.4, Mestrelab Research, Santiago de Compostela, Spain) was employed to measure the *J* value of the peaks, and to identify the peaks of ^1^H-^1^H COSY and ^1^H-^13^C HSQC spectra.

### 2.5. Data Processing and Statistical Analyses

Binning and normalization of ^1^H-NMR spectral data were performed using Chenomx NMR suit software. Baseline corrected NMR spectral data ranging from 0.08 to 10.00 ppm were segmented into a series of small bins (total 245) with widths of 0.04 ppm while excluding the water suppression region (4.70–4.86 ppm). Then the spectra were normalized into total area normalization and standardized area normalization. Intensities in each binned spectral data acquired from total area normalization and standardized area normalization were calculated by relative intensities to the total area of all bins and area of reference peak, respectively. Results of the binned datasets were converted to Microsoft Office Excel (version 2007, Microsoft, Redmond, WA, USA) compatible format to measure each compound by its loading value. Binning values of compounds having multiple non-overlapping peaks were summed. Then, the data was imported into SIMCA (version 15.0, Umetrics, Umeå, Sweden) for (orthogonal) partial least squares-discriminant analysis ((O)PLS-DA) of soybean samples (n = 68). Optimal (O)PLS-DA models were determined by good-fit parameter; R^2^Y and predictability parameter; Q^2^Y, as well as R^2^Y-intercept values and Q^2^Y-intercept values, which were obtained by permutation tests. When establishing the OPLS-DA model, two scaling methods, unit variance (UV) and Pareto, were applied and the results compared to identify the most optimal scaling method. UV scaling and Pareto scaling are methods of dividing each variable by a scaling factor as the standard deviation and square root of standard deviation of the intensity of each metabolite in all samples, respectively. Leave-one-out cross-validation (LOO-CV) was performed to detect and prevent overfitting of the models. LOO-CV leaves out one of the data and the model is built on the remaining data. Then the left out data are predicted repeatedly from the new model until the entire data have been predicted at least once [23].

Sensitivity, specificity, and accuracy were calculated to evaluate the classification performance of the model based on the class prediction value of the sample obtained from the LOO-CV (Y-predcv) by using SIMCA software. Sensitivity is the parameter that measure the classification ability of the model for correct class of cases, whereas specificity measures the prediction ability of the model for correct class of controls [24]. Accuracy means the total proportion of correct class in both cases and controls, which measures the veracity of the model [25]. Receiver operating characteristics (ROC) curve analysis was performed using SIMCA software.

## 3. Results & Discussion

### 3.1. Size Measurement of Soybean Samples

The geographical distribution of the collected Korean and Chinese soybean samples is shown in Figure S1. Soybean samples from each region were photographed (Figure S2), and their sizes were measured. To obtain a representative sample and verify its practical significance, soybeans obtained from local markets were used in the experiments. The average size of Korean soybeans was 8.38 mm, which was significantly larger than that of Chinese soybeans (7.60 mm), as shown in Figure S3A. The size of Chinese soybeans differed widely from region to region, and even from province to province within the same region, whereas the size of Korean soybeans showed no significant regional differences as shown in Figures S2 and S3B. The yield of soybeans was influenced by various factors such as climate, adopted cultural practice, cultivars, and soil conditions [26]. For example, under soil moisture or phosphorous deficiency conditions, the size and number of soybean seeds were markedly decreased, which was a major cause of low soybean yields [27,28]. Therefore, the difference in the size of soybeans between Korea and China or among Chinese regions (and between province and province in the same region) might be affected by differences in the cultivars or environmental factors, such as temperature, precipitation, and soil conditions. As shown in the climate data (Figure S4), there was no significant difference in the average annual temperature and precipitation between Korea and China. However, in China, the average annual temperature and precipitation were significantly different among the three regions. When comparing the climate data of each province in China, the average annual precipitation was significantly different by province only for the middle region; however, the average annual temperature was not different among provinces in all respective regions.

The large-seeded species might have an advantage during seedling establishment, whereas the small-seeded species might have an advantage during seed production under various environmental conditions [29]. Although cultivar information in both countries could not be obtained for the present study, the selection of larger soybean varieties might have occurred since ancient times in Korea [30]. Hence, it is assumed that this tendency has been ongoing and has resulted in a larger size of representative soybean cultivars in Korea than in China.

### 3.2. Identification of Soybean Metabolites Using NMR Spectroscopy

Representative ^1^H-NMR spectra of Korean and Chinese soybeans are shown in Figure S5. We obtained 68 ^1^H-NMR spectra using four experimental replications from eight and nine soybean samples from Korea and China, respectively. A total of 25 metabolites were identified, as listed in Table 1. These included ten amino acids (alanine, asparagine, aspartate, choline, galactarate, glutamate, isoleucine, leucine, tryptophan, and valine), three carbohydrates (glucose, raffinose/stachyose, and sucrose), nine organic acids (2-oxoglutarate, acetate, acetoacetate, citrate, formate, fumarate, malonate, succinate, and tartarate), one fatty acid (2-hydroxyisobutyrate), and others (hypoxanthine and oxy-purinol). Using ^1^H-^1^H COSY and ^1^H-^13^C HSQC spectral data, 16 and 23 metabolites were confirmed, respectively (Figure S6).

### 3.3. Discrimination and Prediction of Korean and Chinese Soybean Samples

The selection of the most appropriate normalization and scaling methods is an important step for improving the biological information of metabolomics data as it decreases any unwanted biases originating from biological and technical variance and adjusts different ranges between samples or variables for comparison [31,32]. Normalization is a row operation that reduces the significant intensity variation of metabolites between samples (sample-to-sample variation); therefore, all samples can be compared with each other. Scaling is a column operation that adjusts the intensity variance between metabolites (metabolite-to-metabolite), and thus, all metabolites can be compared with each other [33].

OPLS-DA and permutation tests were performed to determine the optimal normalization (standardized area normalization versus total area normalization) and scaling methods (UV versus Pareto) to discriminate between Korean and Chinese soybeans. The number of components was selected using the autofit function in SIMCA software, which extracts a significant number of principal components from the models. The optimal model was determined by a good-fit parameter, R^2^Y, and a predictability parameter, Q^2^Y, with values close to 1, which indicate good fitness and prediction of the experiment, respectively.

The R^2^Y- and Q^2^Y-intercept values from permutation tests should be below 0.40 and 0.05, respectively, in a statistically valid OPLS-DA model [34]. As listed in Table 2, the highest R^2^Y value of 0.882 and Q^2^Y value of 0.783 were obtained by applying the total area normalization, UV scaling, and five OPLS components (1+4) with satisfactory R^2^Y- and Q^2^Y- intercept values of 0.254 and –0.487, respectively. The R^2^Y-intercept value of 0.254 and Q^2^Y-intercept value of –0.487 from the permutation test proved the statistical validity of the model without overfitting of the data. The total area normalization used in the model for discriminating between Korean and Chinese soybeans divided each metabolite peak area by the total peak area; therefore, each sample had the same total peak area unit of 1. This is one of the most commonly used normalization methods in NMR metabolomics. Thus, each peak intensity can be expressed as a fraction of the total peak intensity (as a percentage), making it possible to compare each metabolite level between samples in the same unit [33]. The UV scaling method is one of the easiest ways to adjust the metabolic variations by giving each metabolite the same importance, making the standard deviation equal to 1 for all metabolites [35,36].

Based on the selected total area normalization and UV scaling methods, various variable influences on projection (VIP) values were applied to select the optimal OPLS-DA model. The VIP values from the 25 metabolites are listed in Table S2. As listed in Table S3, the best OPLS-DA model (R^2^Y 0.882 and Q^2^Y 0.783) for discriminating between Korean and Chinese soybean samples was constructed without applying the VIP cut-off value.

**Figure 1:**
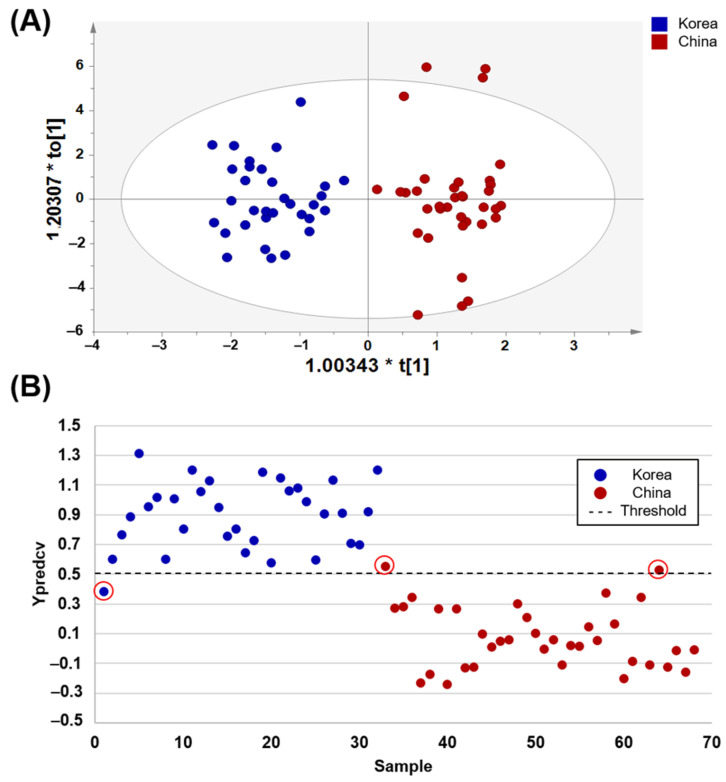
OPLS-DA score plots (**A**) derived from the ^1^H-NMR spectra of soybean samples for discriminating geographical origin, Korea and China. Leave-one-out cross-validated score plots (**B**) showing Korean soybeans (above dashed line) and Chinese soybeans (below dashed line) with threshold value of 0.5 (dashed line) for all samples. In case of misclassified samples, the Ypredcv values were marked with red circles.

OPLS-DA-derived score plots (Figure 1A) showed clear discrimination between Korean and Chinese soybean samples. Y prediction plots using the LOO-CV are shown in Figure 1B. These were used to evaluate the ability of the OPLS-DA model to determine the origin of soybean samples (i.e., Korea or China). A threshold value of 0.5 was adopted to classify the origin of the soybean samples. Except for one Korean sample (Kangwon-Yeongwol) and two Chinese samples (Heilongjiang and Guangdong provinces), most of the samples were classified correctly, showing 96.9% sensitivity, 94.4% specificity, and 95.6% accuracy (Table 3). ROC curve analysis showed the area under the curve (AUC) value of 1.0 for predicting the geographical origin of soybean samples from Korea and China (Figure S7A). This identified 25 metabolites which could be used as potential biomarkers differentiating the geographical origin of soybean sample from Korea and China. Thus, the OPLS-DA model obtained in the present study is useful for discriminating and predicting Korean and Chinese soybean samples.

The relative levels of metabolites in the soybean samples from Korea and China are listed in Table S4. Among the 25 total metabolites, nine showed significantly different relative levels when comparing Korean and Chinese soybean samples. Relative levels of alanine, citrate, isoleucine, tartarate, and valine were significantly higher (*p* < 0.05) in Korean soybeans than Chinese soybeans, whereas those of asparagine, choline, galactarate, and tryptophan were significantly higher in Chinese soybeans than Korean soybeans. These nine metabolites (VIP value over 0.87) were suggested as influential contributors to the differentiation between Korean and Chinese soybean samples (Table S2).

Furthermore, this study investigated the possibility of a discriminant model for soybean samples from Korea and northeastern China. In geographic coordinates, these two sites are located relatively close along the latitudinal and longitudinal axis (38° N, 127° E in Korea, 41–48° N, 122–129° E in northeastern China). As shown in Figure S8A,B, soybean samples from Korea and northeastern China were well discriminated. The LOO-CV test showed an accuracy of 100% for predicting origin (Figure S8C). Accordingly, soybean samples from Korea and northeastern China could also be differentiated by NMR-based metabolic profiling, even though the geographical locations of Korea and northeastern China are relatively close.

Differences in the metabolic profile levels of seed plants including soybeans are influenced by genetic factors, such as cultivar; however, they are also influenced by environmental factors [37,38]. Based on thousands of years of the rich planting experience of breeders and on specific environmental conditions, many scientists and breeders in each region have developed their own environmentally compatible soybean varieties, such as drought-, cold-, disease-, and insect-resistant varieties, to accommodate environmental conditions in soybean breeding programs or using historical landraces maintained by farmers for their seed lots. Therefore, the influence of the variety and environmental background factors on differences in metabolites between Korean and Chinese soybean samples could be considered. Korea and China are the main countries that have a long cultivation history and broad genetic diversity of soybean, and soybean genotypes collected from these countries are known to be different [39,40,41,42]. The history of soybean cultivation in Korea began 2500 years ago, and different cultivars with various forms and agronomic characteristics were formed over the years, which were utilized as genetic resources for the improvement of modern varieties. However, these native soybeans were quickly replaced by newly developed varieties, which lowered the genetic diversity of Korean soybean varieties [43]. The history of soybean cultivation in China began 3000 years ago, and the northern, Huang Huai (area between Yellow River–Huai Rivers) and southern regions are the major cultivation areas [44]. China has a diverse climate with varying soil characteristics and rainfall patterns. Over 1400 soybean cultivars have been developed in China between 1923 and 2013 [45] through a scientific breeding program; therefore, the parents of the cultivars were landraces, cultivars, breeding lines, and exotic introductions. During the past 20 to 40 years, the yield level of soybean cultivars has increased from 750 kg/hm^2^ to 2000 kg/hm^2^ in China [46].

Although we could not obtain cultivar information for soybeans, the significantly different relative levels of various metabolites between Korean and Chinese soybeans might be due to different environmental conditions, such as temperature, precipitation, and soil conditions (type, particle size, and nutrient content), which are closely related to metabolic expression. The growth and metabolic characteristics of the same cultivar varied depending on the environmental conditions of the growing region, which caused differences in its cellular metabolism [47].

There are several studies reporting the effect of various soil conditions on the yield and quality characteristics of soybean seeds. Higher soybean seed yield was observed in soil with low phosphorous and potassium content, and the yield was also improved under the physical properties of soil with a higher clay content, which was due to the high plant-available water in that type of soil during the growing season [48]. Soybean seed composition varied depending on the soil nutrient content. In the correlation analysis of soil nutrients and seed composition, low contents of soil organic matter and elements such as N, C, K, B, and Zn were related to the lower content of soybean protein and oleic acid [49]. Similarly, amino acid content in soybean was positively correlated with the contents of soil elements such as B, Mo, Se, K and N [50]. Soil moisture stress is also an important abiotic factor for modulating the soybean yield and quality. Under a deficient soil moisture condition, soybean seed yield was reduced and the contents of total protein, palmitic acid, linoleic acid, sucrose, raffinose, stachyose, N, P, K and Ca were markedly decreased, whereas those of total oil, stearic acid, oleic acid, linolenic acid, Fe, Mg, Zn, Cu and B were increased [51]. Therefore, it is suggested that the contents of soil nutrients or moisture during the soybean reproductive periods could be critical factors for influencing the yield and nutrient value (various metabolites) in soybean seeds.

In our study, there was no significant difference in the average annual temperature and precipitation of Korea and China (Figure S4), so it is assumed that the soil moisture or nutrient contents might be different depending on the soil conditions in each country. Even with the same precipitation environment, soil moisture content could be different according to various types, textures, and particle size of soils in each region or country, because the moisture-holding capacity could differ depending on the soil condition. Therefore, other environmental factors, such as soil conditions, rather than temperature and precipitation, and diverse soybean cultivars improved over centuries to adapt to the particular environmental conditions of each region, might be important factors influencing the different levels of metabolites in Korean and Chinese soybeans.

### 3.4. Discrimination and Prediction of Domestic Chinese Soybean Samples

Korean soybeans could not be categorized (data not shown) according to various provinces, possibly because most of the provinces in Korea have similar climatic, soil, and rainfall conditions as well as soybean varieties grown on a small land scale. Therefore, we focused on the differentiation in domestic Chinese soybean samples.

Various normalization and scaling methods were tested to determine the optimal PLS-DA model to differentiate domestic Chinese soybean samples. The highest R^2^Y value of 0.898 and Q^2^Y value of 0.651 were obtained by applying standardized area normalization, UV scaling, and six PLS components satisfying R^2^Y and Q^2^Y intercept values in the PLS-DA model (Table 4). Standardized area normalization divides the intensity of each metabolite by a constant concentration of the internal standard compound, allowing the measurement of the contribution of each metabolite to the spectrum [52]. Because internal standards added prior to extraction can monitor and correct the intensity drift that might occur during extraction and instrument analysis, standardized area normalization can reduce the difference in extraction efficiency between samples [53,54]. Meanwhile, the Pareto scaling method emphasizes weak peaks with high biological relevance and reduces the effect of intense peaks, thereby reducing the effect of noise variables more than the UV scaling method [55].

Based on the selected standardized area normalization and UV scaling methods, various VIP cut-off values were applied to select the optimal OPLS-DA model. The VIP values of the 25 metabolites are listed in Table S5. When a VIP cut-off value of 1.0 was applied, the best PLS-DA model was obtained with 11 metabolites showing the highest R^2^Y value of 0.887, Q^2^Y value of 0.789, R^2^Y intercept value of 0.151, and Q^2^Y intercept value of –0.480 (Table S6).

**Figure 2:**
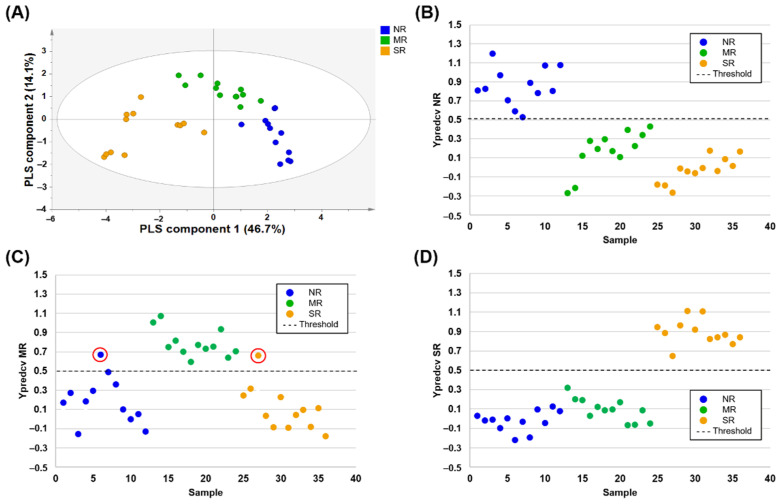
PLS-DA score plots (**A**) derived from the ^1^H-NMR spectra of soybean samples for discriminating the geographical origin of three regions of China. Leave-one-out cross-validated score plots for NR vs. MR/SR comparative group (**B**), MR vs. NR/SR comparative group (**C**), and SR vs. NR/MR comparative group (**D**) showing control sample (one region, above dashed line) and case sample (two regions, below dashed line) with threshold value of 0.5 (dashed line) for all samples. In case of misclassified samples, the Ypredcv values were marked with red circles. NR: northeastern region, MR: middle region (Huang-Huai-Hai and Yangtze River region), SR: southern region.

Chinese soybean samples from the northeastern region (NR), middle region (MR), and southern region (SR) were successfully differentiated by NMR-based metabolic profiling (Figure 2A). In the development of the PLS-DA model for discriminating between more than two groups, cross-validation could be conducted in three groups that were binary coded with the three dummy Y variables, (0 1 1) for samples in class 1, (1 0 1) for samples in class 2, and (1 1 0) for samples in class 3, where 0 and 1 indicate the control and case, respectively [56]. Therefore, three independent class approaches were employed in our study. Y prediction plots after performing the LOO-CV are shown in Figure 2B–D. When designating control groups as both NR and SR independently, Ypredcv values (predicted Y values of each left-out sample calculated after performing cross-validation) of each control and case groups were correctly classified with a threshold value of 0.5 (Figure 2B,D). Only two misclassified samples were detected in the model designating the MR to the control group (Figure 2C). In summary, 100% sensitivity, specificity, and accuracy were obtained in the model designating the NR and SR to the control group, and 100% sensitivity, 91.7% specificity, and 94.4% accuracy were obtained in the model designating the MR to the control group (Table 3).

In ROC curve analysis, all AUC values for predicting the origin of soybean samples from three Chinese regions were 1.0 (Figure S7B–D). It was confirmed that the selected 11 metabolites could be used as potential biomarkers for the differentiation of geographical origin of soybean samples from three Chinese regions.

Seventeen metabolites showed significantly different levels among soybeans from the three regions of China analyzed in our study (Table S7). Among these 17 metabolites, four metabolites (glucose, hypoxanthine, leucine, and tartarate) showed significantly higher levels in the NR, and SR was characterized by significantly higher levels of malonate alone. However, in the MR, no particular metabolite showed a relatively higher level than the other regions.

The present study assumed that the differences in soybean metabolites among Chinese regions might be affected by different environmental conditions including temperature, precipitation, and soil as well as cultivar differences. Based on China’s regional climate statistics, there were significant differences in the average annual temperature and total precipitation among the three Chinese regions in 2016 (Figure S4). The average annual temperature in the NR of China was the lowest compared to the other regions, and there was no significant difference in temperature between the MR and SR. The average annual precipitation was the highest in the SR of China compared to the other regions, whereas no significant difference was found in the average annual precipitation between the NR and MR. Sugar accumulation has been observed in soybeans obtained from the cold northeastern region of China, whereas amino acid and total protein content was abundant in soybeans sourced from the warm southern region in China [38]. North-to-south latitudinal gradients in China have a greater influence on metabolite profile changes in soybeans than east-to-west longitudinal gradients [38]. This is consistent with our study results showing the lowest temperature and highest soybean glucose level, which are characteristic features of soybeans from northeastern China. Plants exposed to low temperatures accumulate starch-derived sugars to adapt to cold stress and use sugars as an energy source or cryoprotectant [57].

Previous studies have reported that the accumulation of alanine and gamma-aminobutyric acid (GABA) in soybean roots is the most characteristic response to flood stress [58]. This is consistent with our findings that soybean alanine levels were the highest in the SR of China, which had relatively higher precipitation than the other regions. The accumulation of amino acids such as alanine and GABA is an important adaptation mechanism for storing insufficient carbon and nitrogen in an oxygen-deficient environment [58]. Therefore, flood-stressed plant cells maintain intracellular osmotic pressure by accumulating amino acids to compensate for the reduced soluble carbohydrate level [58].

In the present study, Chinese soybeans could be differentiated based on their cultivation locations (i.e., northeastern, middle, and southern regions), suggesting that regionally adapted varieties were characterized in each region of China under their own inherent growing environments. We previously reported the classification of soybeans from Korea and China using FT-IR spectroscopy [59]. The NMR-based differentiation and prediction methods for identifying the geographical origin of soybeans in the present study could be used as a complementary method to the FT-IR-based technology to provide orthogonal criteria for more precisely discriminating soybean samples.

## 4. Conclusions

In the present study, NMR spectroscopy coupled with multivariate statistical analysis was employed to differentiate the geographical origin of soybeans. We succeeded in discriminating and predicting the origin of Korean and Chinese soybeans using the OPLS-DA model with LOO-CV. In addition, soybeans from the northeastern, middle, and southern regions of China were differentiated using PLS-DA models. Based on the requirements of growers and consumers, various soybean varieties have been planted and grown worldwide. These soybean varieties have been utilized in soybean products such as soy oil, soy sauce, tofu, and soymilk, and the market life of soybean varieties is short. Hence, prediction models and databases for investigating the geographical origin of soybeans should be regularly updated (at least every 2–3 years). As an investigative work, we established a simple, efficient, and convenient method for discriminating the precise geographical origin of soybeans in the present study. This method could be widely used for the detection of soybean samples with intentionally mislabeled geographical origin. In future studies, soybean samples from diverse geographical locations worldwide that have been harvested in different years will be investigated using the methods developed in the present study.

## Figures and Tables

**Table 1:** Assignment of ^1^H-NMR (nuclear magnetic resonance) spectral peaks of soybean samples.

No.	Compounds	Chemical Shift	Multiplicity; *J* Value	Assignment	Assignment Method
1	2-hydroxyisobutyrate	1.34	s	H-3, H-4	One-dimensional proton NMR (1D)/heteronuclear single quantum correlation (HSQC)
2	2-oxoglutarate	2.44	t; *J* = 6.92	H-5	1D/correlation spectroscopy (COSY)/HSQC
		2.99	t; *J* = 6.92	H-4	
3	Acetate	1.91	s	H-2	1D/HSQC
4	Acetoacetate	2.28	s	H-4	1D/HSQC
5	Alanine	1.47	d; *J* = 7.19	H-3	1D/COSY/HSQC
		3.78	q; *J* = 7.19	H-2	
6	Asparagine	2.82–2.88	m	H-2	1D/COSY/HSQC
		2.90–2.98	m	H-2	
		4.01	q; *J* = 4.26	H-3	
7	Aspartate	2.62	dd; *J* = 8.7, 14.43	H-2	1D/COSY/HSQC
		2.79	dd; *J* = 3.78, 13.68	H-2	
		3.91	dd; *J* = 3.75, 4.92	H-3	
8	Choline	3.20	s	H-3, H-4, H-5	1D/COSY/HSQC
		3.48–3.53	m	H-2	
		4.03–4.09	m	H-1	
9	Citrate	2.54	d; *J* = 15.36	2Ha, 4Ha	1D/COSY/HSQC
		2.68	d; *J* = 15.36	2Hb, 4Hb	
10	Formate	8.46	s	H-1	1D
11	Fumarate	6.52	s	H-2, H-3	1D
12	Galactarate	3.94	s	H-3, H-4	1D/COSY/HSQC
		4.26	s	H-2, H-5	
13	Glucose	3.22	dd; *J* = 1.44, 7.95	H-2	1D/COSY/HSQC
		3.38–3.43	m	H-4	
		3.48–3.54	m	H-5	
		3.52	dd; *J* = 3.7, 9.82	H-2	
		3.72–3.78	m	H-3, H-6	
		3.80–3.85	m	H-5, H-6	
		4.62	d, *J* = 7.92	H-1	
		5.22	d; *J* = 3.72	H-1	
14	Glutamate	2.00–2.08	m	H-3	1D/COSY/HSQC
		2.10–2.18	m	H-3	
		2.28–2.40	m	H-4	
		3.75	dd; *J* = 4.8, 2.4	H-2	
15	Hypoxanthine	8.17	s	H-2	1D/COSY/HSQC
		8.20	s	H-8	
16	Isoleucine	0.93	t; *J* = 7.15	H-5	1D/COSY/HSQC
		1.00	d; *J* = 7.15	CH_3_	
		1.41–1.49	m	H-4	
		1.92–2.01	m	H-3	
		3.66	d; *J* = 4.08	H-2	
17	Leucine	0.94	t; *J* = 6.06	H-5, CH_3_	1D/COSY/HSQC
		1.64–1.78	m	H-3, H-4	
		3.69–3.76	m	H-2	
18	Malonate	3.13	s	H-2	1D/HSQC
19	Oxypurinol	8.27	s	H-7	1D/HSQC
20	Raffinose/Stachyose	3.52	t; *J* = 4.5	H-4′	1D/COSY/HSQC
		3.69	br. s	H-6	
		3.95	t; *J* = 6.36	H-5″	
		4.95	dd; *J* = 2.7, 4.1	H-1″	
		5.41	d; *J* = 4.5	H-1	
21	Succinate	2.42	s	H-2, H-3	1D/HSQC
22	Sucrose	3.55	dd; *J* = 3.84, 6.12	H-1′	1D/COSY/HSQC
		3.66	s	H-1	
		3.75	t; *J* = 9.05	H-3	
		3.76–3.84	m	H-6	
		4.04	t; *J* = 9.05	H-4′	
		5.39	d; *J* = 3.84	H-1	
23	Tartarate	4.34	s	H-2, H-3	1D/HSQC
24	Tryptophan	7.20–7.24	m	H-8	1D/COSY/HSQC
		7.18–7.28	m	H-9	
		7.32	s	H-2	
		7.71	d; *J* = 7.92	H-7	
25	Valine	0.98	d; *J* = 7.02	CH_3_	1D/COSY/HSQC
		1.05	d; *J* = 7.14	H-4	
		2.20–2.32	m	H-3	
		3.61	d; *J* = 4.33	H-2	

s, singlet; d, doublet; dd, doublet of doublet; t, triplet; q, quartet; m, multiplet; br, broad.

**Table 2:** Parameters of orthogonal partial least squares-discriminant analysis (OPLS-DA) models based on various normalization and scaling methods to discriminate between Korean and Chinese soybean samples.

Group No.	Normalization Method	Scaling Method	Number of Component	R^2^Y	Q^2^Y	R^2^Y Intercept	Q^2^Y Intercept
1	Standard	UV	4	0.844	0.762	0.218	−0.430
2		Par	7	0.861	0.779	0.197	−0.417
3	Total	UV	5	0.882	0.783	0.254	−0.487
4		Par	6	0.862	0.798	0.165	−0.385

Number of components obtained from autofit function in SIMCA software; OPLS-DA, orthogonal partial least squares discriminant analysis; Standard, standardized area normalization; Total, total area normalization; UV, unit variance; Par, Pareto; The bold characters indicate the selected optimal model parameters.

**Table 3:** Classification performance parameters (sensitivity, specificity and accuracy) of OPLS-DA model to discriminate between Korean and Chinese soybeans, and partial least squares-discriminant analysis (PLS-DA) models for discriminating between three regions for Chinese soybeans using leave-one-out cross-validation.

Class		Sensitivity %	Specificity %	Accuracy %
Korea vs. China		96.9	94.4	95.6
China	NR vs. MR&SR	100.0	100.0	100.0
	MR vs. NR&SR	100.0	91.7	94.4
	SR vs. NR&MR	100.0	100.0	100.0

NR: northeastern region, MR: middle region (Huang-Huai-Hai and Yangtze River region), SR: southern region.

**Table 4:** Parameters of PLS-DA models based on various normalization and scaling methods to discriminate between the different origins of Chinese soybean samples.

Group No.	Normalization Method	Scaling Method	Number of Component	R^2^Y	Q^2^Y	R^2^Y Intercept	Q^2^Y Intercept
1	**Standard**	**UV**	**6**	**0.898**	**0.651**	**0.348**	**−0.821**
2		Par	2	0.492	0.348	0.082	−0.189
3	Total	UV	3	0.731	0.622	0.197	−0.345
4		Par	4	0.713	0.566	0.142	−0.468

Number of components obtained from autofit function in SIMCA software; PLS-DA, partial least squares discriminant analysis; Standard, standardized area normalization; Total, total area normalization; UV, unit variance; Par, Pareto; The bold characters indicate the selected optimal model parameters.

## Data Availability

The data presented in this study are available on request from the corresponding author.

## Supplementary Materials

The following are available online at https://www.mdpi.com/2304-8158/10/2/435/s1, Figure S1: Geographical distribution of Korean and Chinese soybean samples. Map of Korea with 8 provinces of soybean samples (A), Map of China with 3 divided regions of soybean samples consisting 9 provinces (B). Figure S2: Representative pictures of soybean samples from Korea and China. Figure S3: Average size of soybean samples from Korea and China. Comparison of average soybean size in Korea and China (A), Comparison of individual soybean size of each region and province in Korea and China (B). Figure S4: Climate data for soybean cultivation regions in Korea and China in 2016. The average value of monthly mean temperature (A) and total precipitation (B) from January to December. Figure S5: Representative 600 MHz ^1^H-NMR spectra of Korean (A) and Chinese (B) soybean samples. Figure S6: ^1^H-^1^H correlation spectroscopy (COSY, (A)) and ^1^H–^13^C heteronuclear single quantum correlation (HSQC, (B)) spectra of soybean sample. Figure S7: Receiver operating characteristics (ROC) curves and area under the curve (AUC) values for distinguishing geographical origin of soybeans. AUC value of 25 metabolites discriminating soybean samples from Korea and China (A). AUC value 11 metabolites discriminating Chinses soybean samples from NR and MR/SR (B), MR and NR/SR (C), and SR and NR/MR (D). Figure S8: Discrimination model for soybean samples from Korea and northeastern China. OPLS-DA score plots (A) derived from the ^1^H-NMR spectra of soybean samples for discriminating the geographical origin of Korea and northeastern China. Permutation test plots (B) with 100 permutations of OPLS-DA model. Leave-one-out cross-validated score plots (C). Table S1: Products and suppliers’ information of Chinese soybean samples. Table S2: Variable importance projection (VIP) values of metabolites for discriminating Korean and Chinese soybean samples. Table S3: Parameters of OPLS-DA models based on various VIP cut-off values for discriminating Korean and Chinese soybean samples based on total area normalization and UV scaling methods. Table S4: Relative levels of metabolites in Korean and Chinese soybean samples. Table S5: Variable importance projection (VIP) values of metabolites for discriminating different origins of the Chinese soybean samples. Table S6: Parameters of PLS-DA models based on various VIP cut-off values for discriminating different origins of the Chinese soybean samples based on standardized area normalization and UV scaling methods. Table S7: Relative levels of metabolites in Chinese soybean samples.
