# Supplementary material for: Discrimination of the Geographical Origin of Soybeans Using NMR-Based Metabolomics
Source: Foods. 2021 Feb 17;10(2):435. doi: 10.3390/foods10020435 (PMC7922469; doi:10.3390/foods10020435)
Supplement: Supplementary file 1 [file foods-10-00435-s001.pdf]

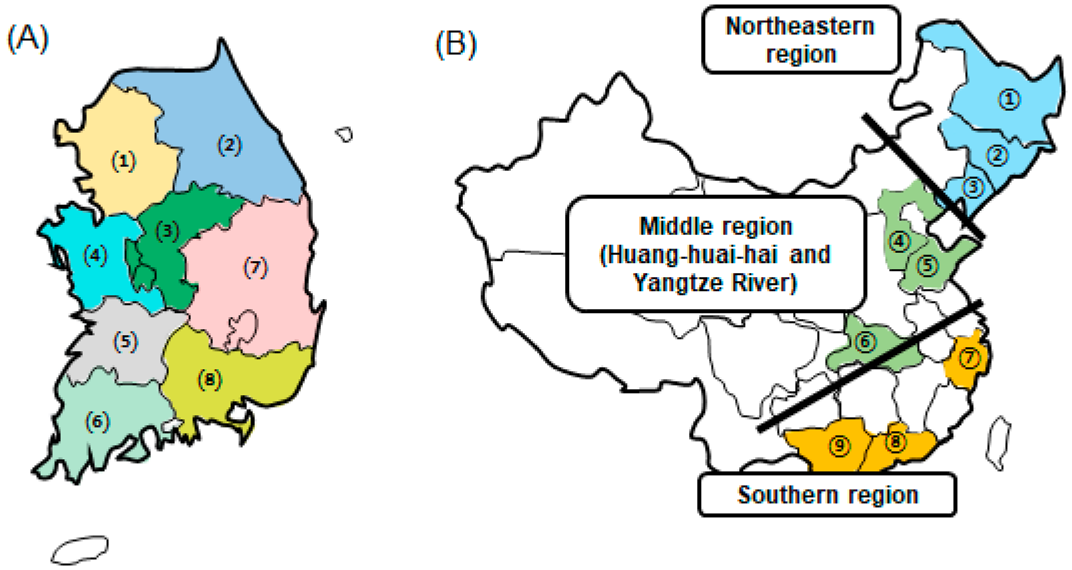

Figure S1. Geographical distribution of Korean and Chinese soybean samples. Map of Korea with 8 provinces of soybean samples (A): (1): Gyeonggi-do, (2): Gangwon-do, (3): Chungcheongbuk-do, (4): Chungcheongnam-do, (5): Jeollabuk-do, (6): Jeollanam-do, (7): Gyeongsangbuk-do, (8) Gyeongsangnam-do; Map of China with 3 divided regions of soybean samples consisting 9 provinces (B): Northeastern region represented with blue color consists of ①: Heilongjiang Province, ②: Jilin Province, ③: Liaoning Province, middle region (Huang-Huai-Hai region and Yangtze River basin region) represented with green color consists of ④: Hebei Province, ⑤: Shandong Province, ⑥: Hubei Province, and southern region represented with orange color consists of ⑦: Zhejiang Province, ⑧: Guangdong Province, ⑨: Guangxi Autonomous Region.

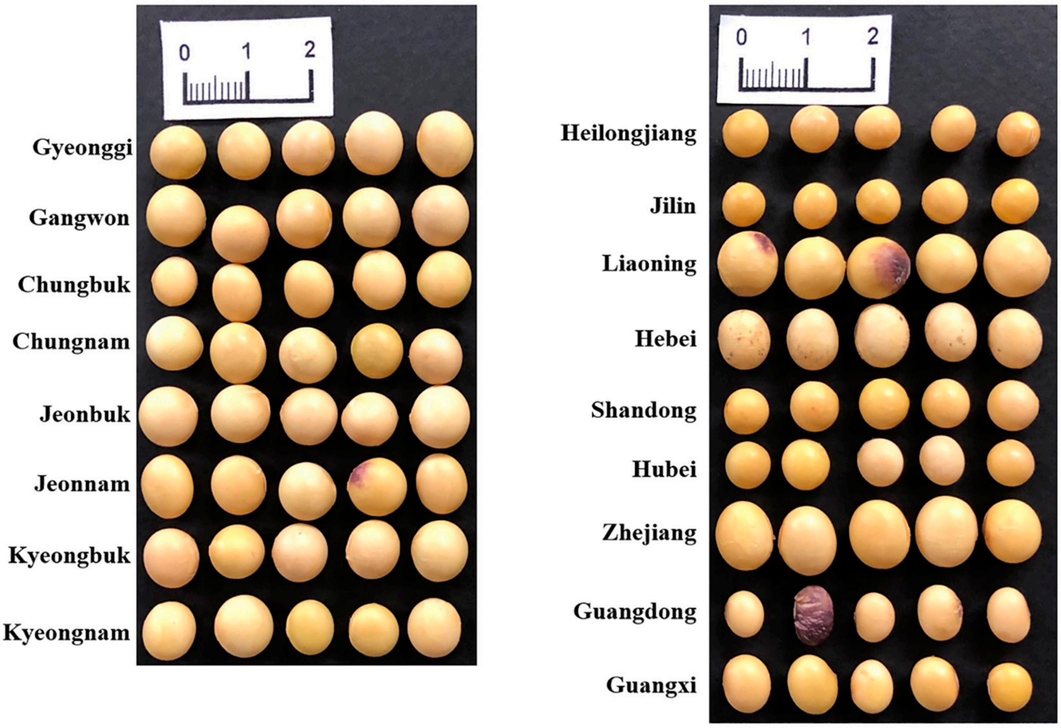

Figure S2. Representative pictures of soybean samples from Korea (left) and China (right).

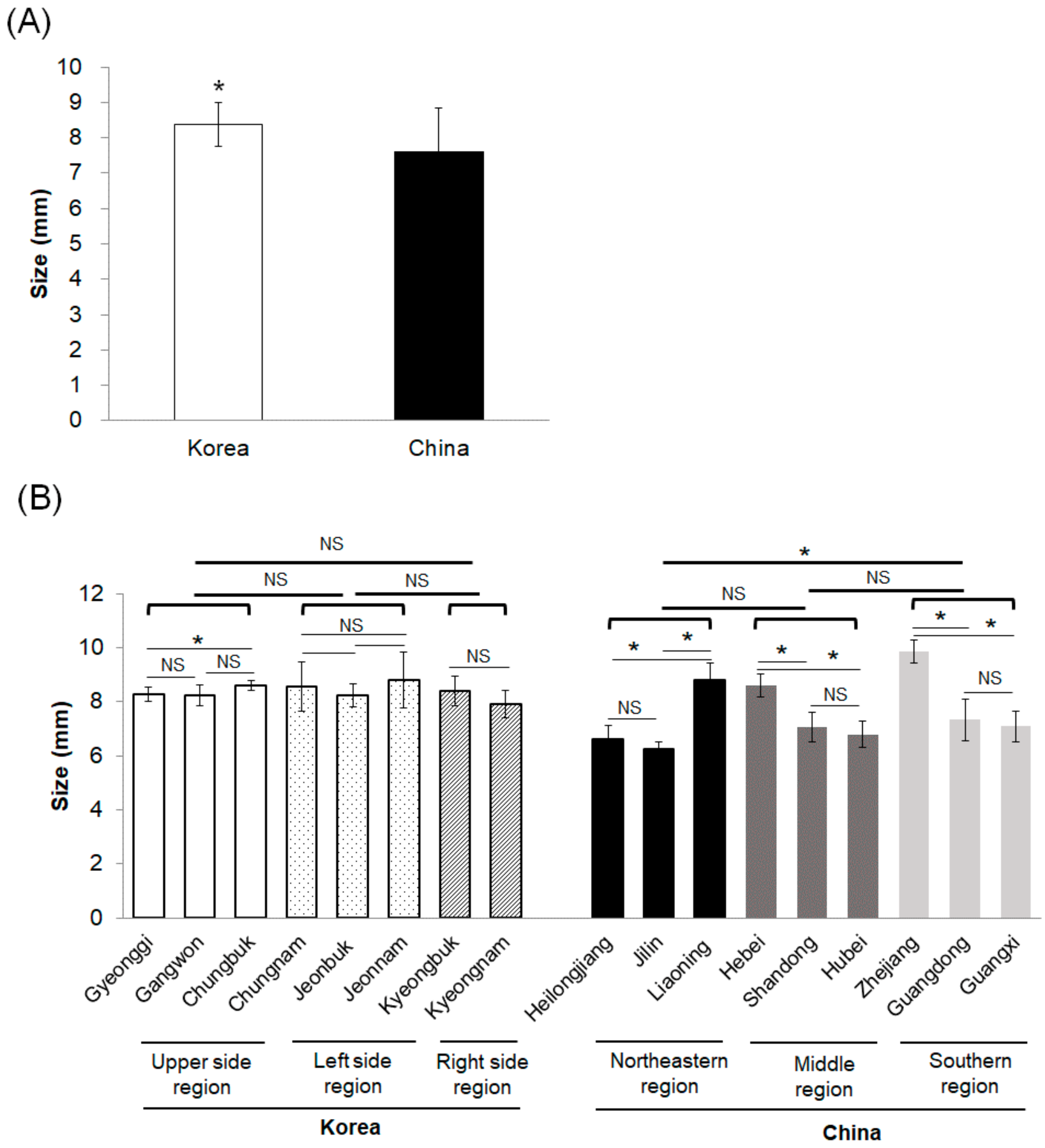

Figure S3. Average size of soybean samples from Korea and China. Comparison of average soybean size in Korea and China (A) determined by independent t-test (p<0.05). Comparison of individual soybean size of each region and province in Korea and China (B). Comparison of regional soybean size in each Korea and China were tested by one-way ANOVA test (p<0.05) and Kruskal-Wallis test (p<0.05) with Bonferroni correction (p<0.017), respectively. Comparison of soybean size from provinces in each Korea and China were tested by Kruskal-

Wallis test (p<0.05) or Mann-Whitney test (p<0.05). The asterisk mark indicates significant differences between samples. NS means not significant. Eight Korean soybean samples of Gyeonggi-do, Gangwon-do, Chungcheongbuk-do, Chungcheongnam-do, Jeollabuk-do, Jeollanam-do, Gyeongsangbuk-do, and Gyeongsangnam-do were abbreviated as Gyeonggi, Gangwon, Chungbuk, Chungnam, Jeonbuk, Jeonnam, Gyeongbuk, and Gyeongnam respectively; nine Chinese soybean samples of Heilongjiang province, Jilin province, Liaoning province, Hebei province, Shandong province, Hubei province, Zhejiang province, Guangdong province, and Guangxi autonomous region were abbreviated as Heilongjiang, Jilin, Liaoning, Hebei, Shandong, Hubei, Zhejiang, Guangdong, and Guangxi respectively.

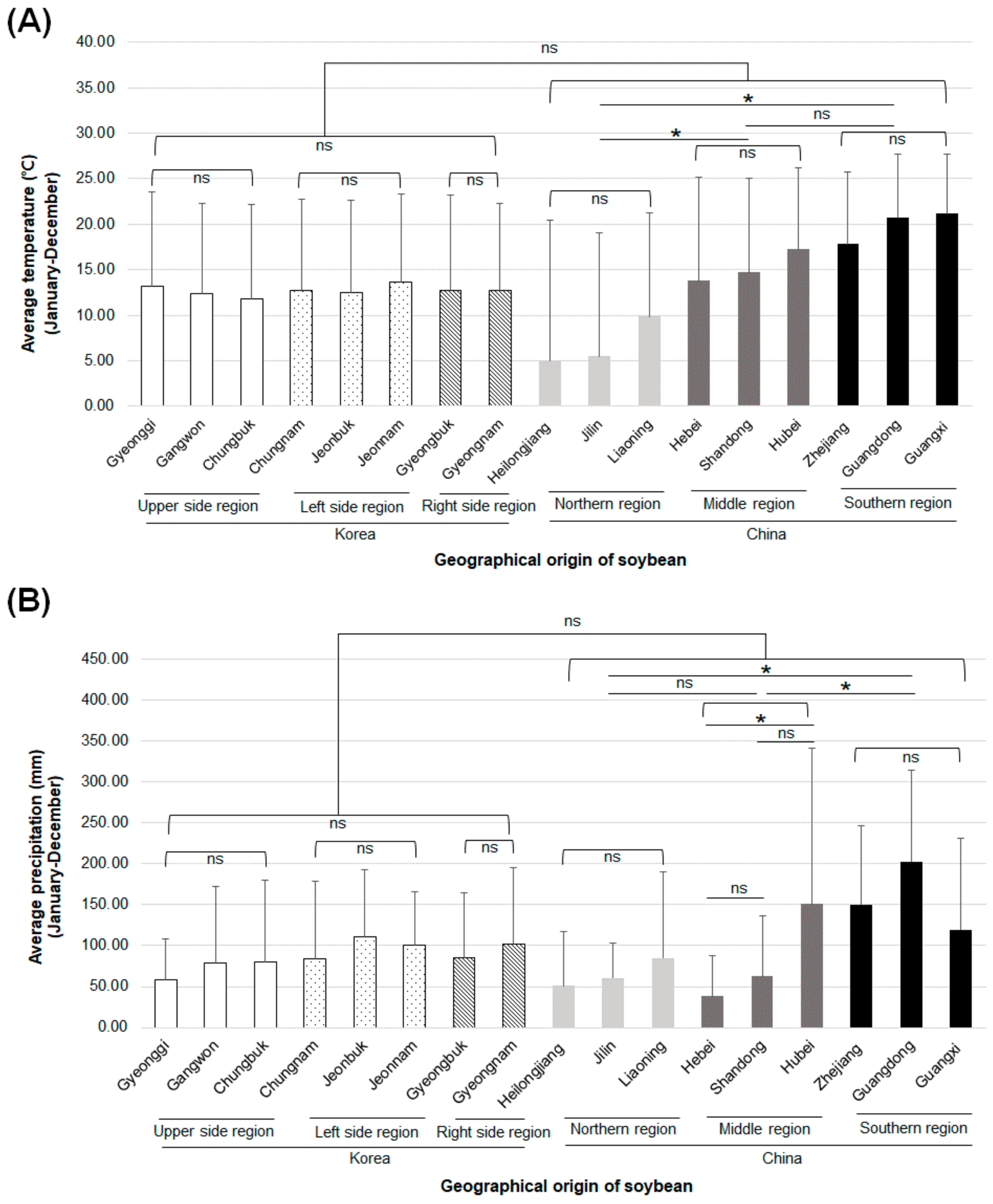

Figure S4. Climate data for soybean cultivation regions in Korea and China in 2016. The average value of monthly mean temperature (A) and total precipitation (B) from January to December. Comparisons of Korea and China was tested by student's t-test (*p*<0.05).

Comparison of regional climate data in each Korea and China were tested by Kruskal-Wallis test ($p<0.05$) and one-way ANOVA test ($p<0.05$), respectively. Comparisons of climate data from provinces in each Korea and China were tested by Kruskal-Wallis test ($p<0.05$) or Mann-Whitney test ($p<0.05$). The asterisk mark indicates significant differences between samples. NS means not significant. Eight Korean soybean samples of Gyeonggi-do, Gangwon-do, Chungcheongbuk-do, Chungcheongnam-do, Jeollabuk-do, Jeollanam-do, Gyeongsangbuk-do, and Gyeongsangnam-do were abbreviated as Gyeonggi, Gangwon, Chungbuk, Chungnam, Jeonbuk, Jeonnam, Gyeongbuk, and Gyeongnam respectively; nine Chinese soybean samples of Heilongjiang province, Jilin province, Liaoning province, Hebei province, Shandong province, Hubei province, Zhejiang province, Guangdong province, and Guangxi autonomous region were abbreviated as Heilongjiang, Jilin, Liaoning, Hebei, Shandong, Hubei, Zhejiang, Guangdong, and Guangxi respectively.

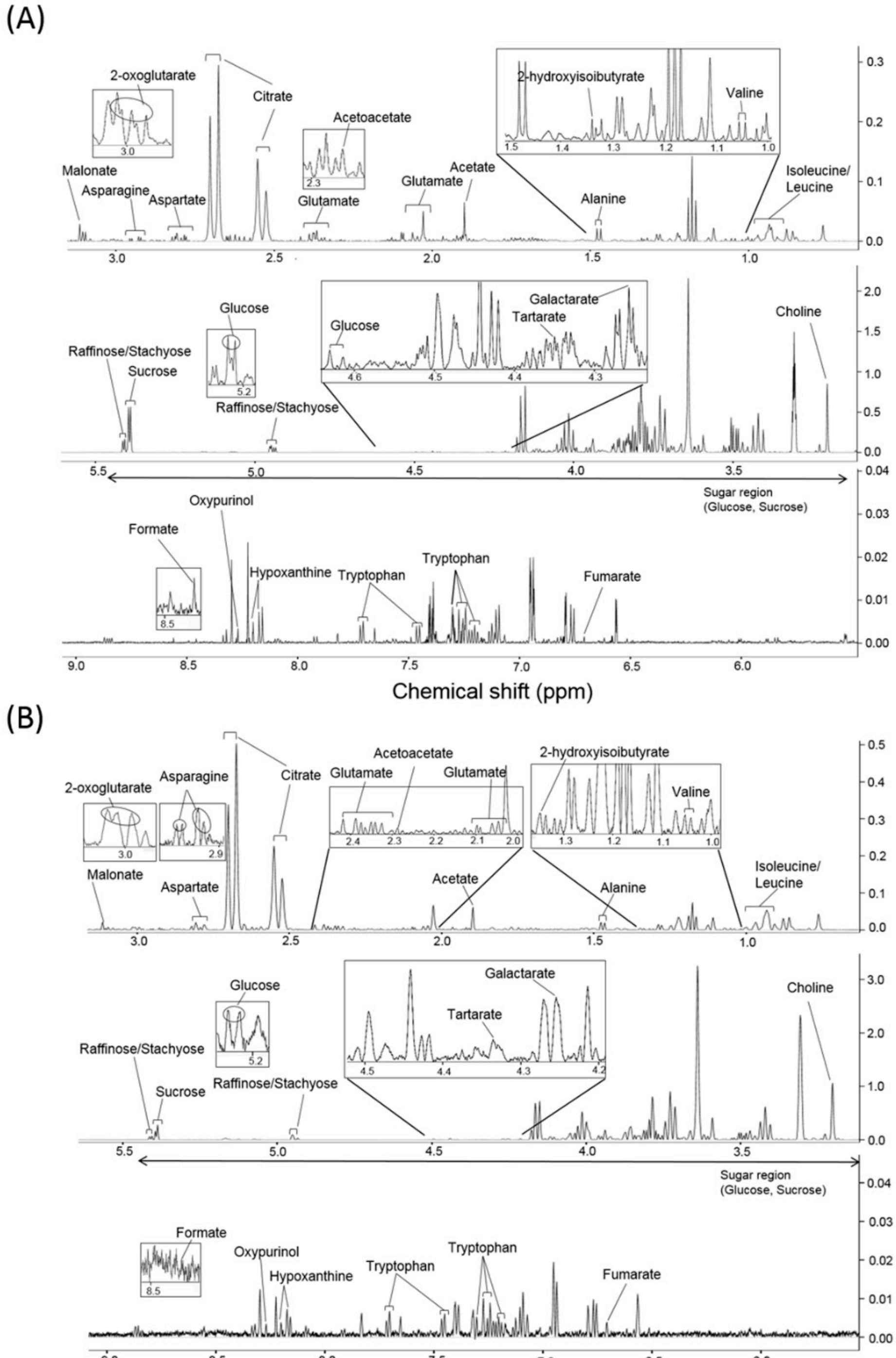

Figure S5. Representative 600 MHz ^{1}H-NMR spectra of Korean (A), and Chinese (B) soybean samples.

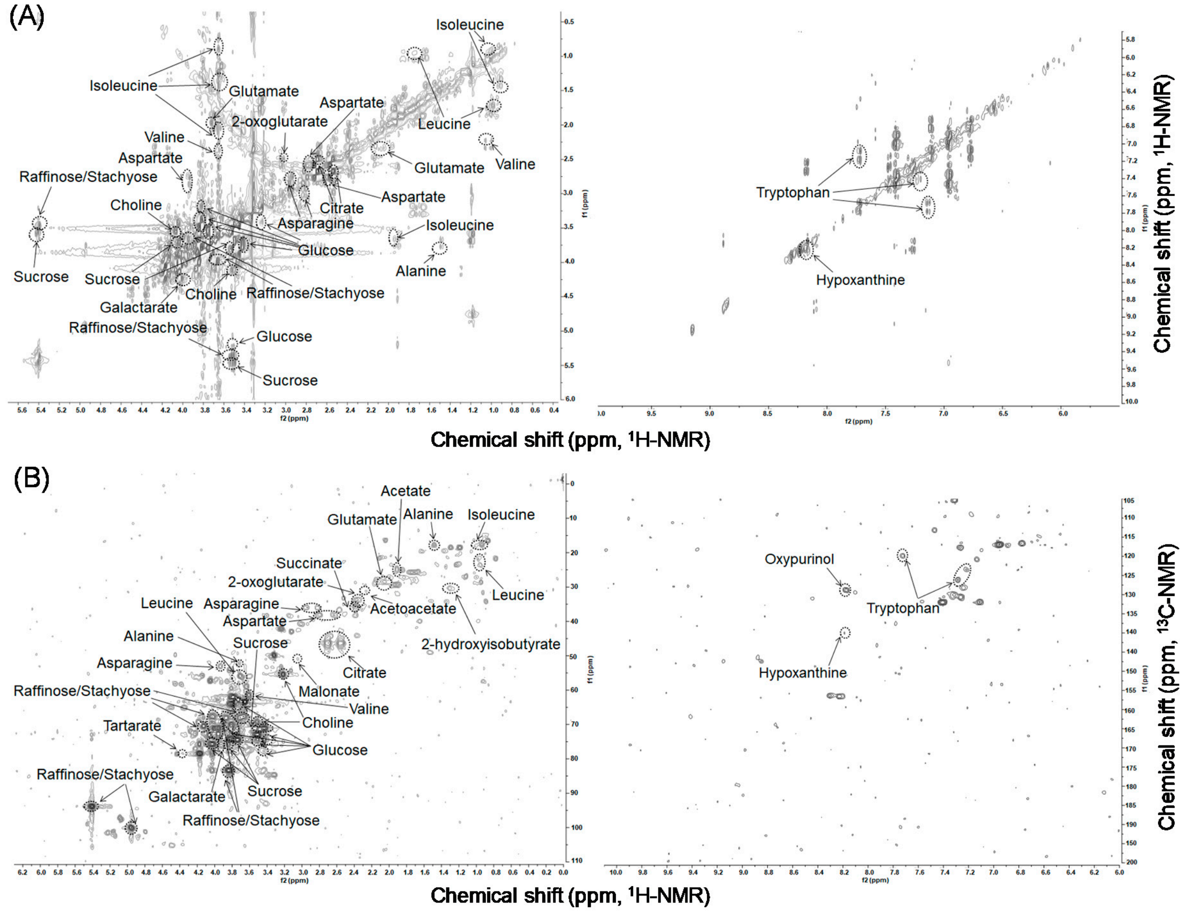

Figure S6. ^{1}H-^{1}H **correlation spectroscopy (COSY, (A)), and** ^{1}H–^{13}C **heteronuclear single quantum correlation (HSQC, (B)) spectra of soybean sample.**

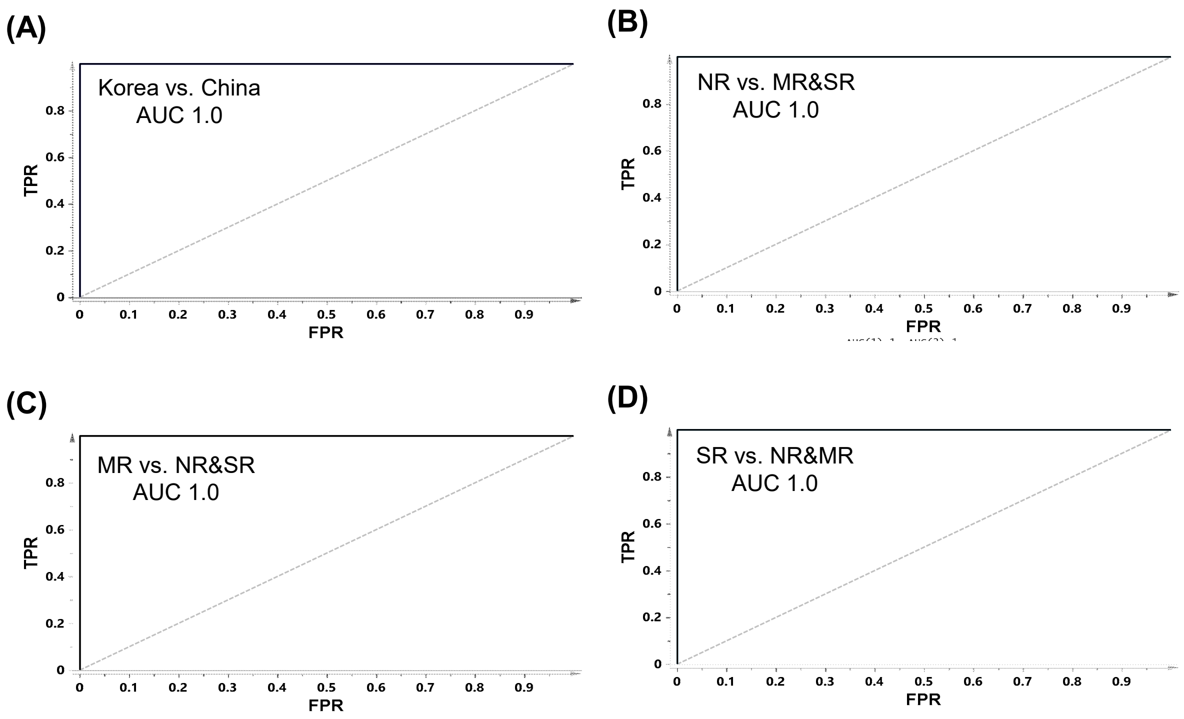

Figure S7. Receiver operating characteristics (ROC) curves and area under the curve (AUC) values for distinguishing geographical origin of soybeans. AUC value of 25 metabolites discriminating soybean samples from Korea and China (A). AUC value 11 metabolites discriminating Chinses soybean samples from NR and MR/SR (B), MR and NR/SR (C), and SR and NR/MR (D). NR: northeastern region, MR: middle region (Huang-Huai-Hai and Yangtze River region), SR: southern region.

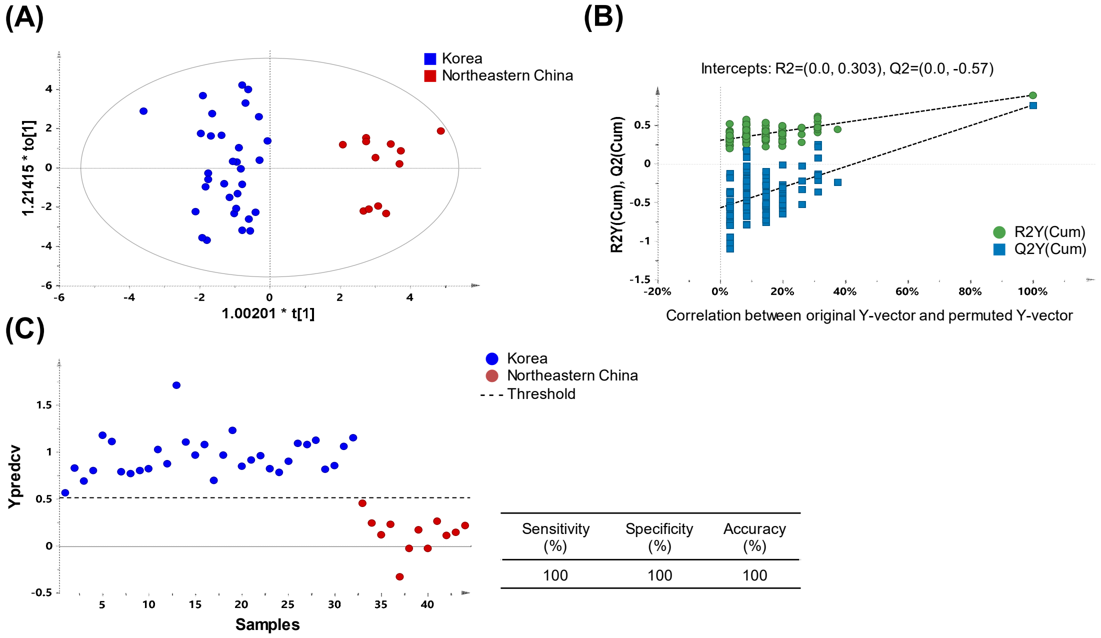

Sensitivity (%)	Specificity (%)	Accuracy (%)
100	100	100

Figure S8. Discrimination model for soybean samples from Korea and northeastern China. OPLS-DA score plots (A) derived from the ^{1}H-NMR spectra of soybean samples for discriminating the geographical origin of Korea and northeastern China. Permutation test plots (B) with 100 permutations of OPLS-DA model. Leave-one-out cross-validated score plots (C) showing Korean soybeans (above dashed line) and northeastern Chinese soybeans (below dashed line) with threshold value of 0.5 (dashed line) for all samples.

Table S1. Products and suppliers' information of Chinese soybean samples

No.	Province	City	Product information		
			Suppliers	Product number	Homepage address
**1**	Heilongjiang	Harbin	Dongbeilaocuinongjiazaliang	None	https://shop118787577.taobao.com/?spm=a1z10.1-c-s.0.0.45aee1ee4CpsWV
**2**	Jilin	Meihekou	Xiaoyaliangpindongbeinongjia	None	https://shop108710895.m.taobao.com/?spm=a1z10.1-c.w4069-7416007653.1.5e6ac960w7GI9y
**3**	Liaoning	Dandong	Shide	QS2106-0102-2907	None
**4**	Hebei	Shijiazhuang	Laoweinongjia	None	https://njlw.taobao.com
**5**	Shandong	Jining	Helaixiangqijiandian	Q / HLX 0002S	http://www.helaixiang.com/
	Hubei	Huangshi	Xiangersaonongjiadian	None	None
**7**	Zhejiang	Pinghu	yanyuqijiapangnongjiadian	None	https://world.taobao.com/dianpu/101949532.htm?spm=a211ha.10565794.0.0.422972d3Q7bB05
**8**	Guangdong	Shaoguan	Wenyueliang	None	None
**9**	Guangxi Zhuang Autonomous Region	Bama Yao Autonomous	Bamayuanwei	None	https://world.taobao.com/item/531284827070.htm?spm=a21wu.11804641-cat-tw.0.0.5f047a67sRYzeA

Table S2. Variable importance projection (VIP) values of metabolites for discriminating Korean and Chinese soybean samples

No.	Compounds	VIP value
1	Tartarate	2.052
2	Galactarate	1.926
3	Valine	1.786
4	Tryptophan	1.754
5	Isoleucine	1.716
6	Citrate	1.375
7	Alanine	1.088
8	Asparagine	0.933
9	Choline	0.877
10	2-Hydroxyisobutyrate	0.875
11	Oxypurinol	0.694
12	Raffinose/Stachyose	0.610
13	Glutamate	0.559
14	Hypoxanthine	0.517
15	Succinate	0.513
16	2-Oxoglutarate	0.404
17	Sucrose	0.391
18	Formate	0.387
19	Aspartate	0.323
20	Malonate	0.220
21	Acetoacetate	0.161
22	Fumarate	0.159
23	Acetate	0.137
24	Leucine	0.073
25	Glucose	0.003

Table S3. Parameters of OPLS-DA models based on various VIP cut-off values for discriminating Korean and Chinese soybean samples based on total area normalization and UV scaling methods

VIP cut-off	Number of variable	Number of component	R^2Y	Q^2Y	R^2Y intercept	Q^2Y intercept
**Total area normalization, UV scaling**						
**0**	**25**	**5**	**0.882**	**0.783**	**0.254**	**-0.487**
>0.8	10	2	0.690	0.620	0.062	-0.198
>0.9	8	3	0.695	0.627	0.055	-0.191
>1.0	7	3	0.688	0.621	0.036	-0.181
>1.5	5	2	0.659	0.606	0.003	-0.139

Number of components obtained from autofit function in SIMCA software; VIP, variable importance projection; UV, unit variance; The bold characters indicate the selected optimal model parameters.

Table S4. Relative levels of metabolites in Korean and Chinese soybean samples (*, $p < 0.05$)

No.	Compounds	Korea	China
1	Acetate	$2.55±0.83^{NS}$	2.40±2.41
2	Acetoacetate	$0.35±0.10^{NS}$	0.34±0.18
3	Alanine	0.75±0.32*	0.51±0.40
4	Asparagine	1.09±0.62*	1.75±1.61
5	Aspartate	$2.57±0.94^{NS}$	2.85±1.57
6	Choline	85.91±5.60*	89.00±5.66
7	Citrate	58.09±9.24*	50.09±10.33
8	Formate	$0.09±0.02^{NS}$	0.10±0.02
9	Fumarate	$0.14±0.02^{NS}$	0.14±0.03
10	Galactarate	1.05±0.36*	1.41±0.22
11	Glucose	$0.58±0.15^{NS}$	0.58±0.22
12	Glutamate	$7.28±0.80^{NS}$	7.09±0.80
13	2-Hydroxyisobutyrate	$0.87±0.31^{NS}$	0.99±0.22
14	Hypoxanthine	$0.47±0.11^{NS}$	0.51±0.19
15	Isoleucine	0.65±0.14*	0.50±0.17
16	Leucine	$4.20±0.93^{NS}$	4.29±0.75
17	Malonate	$0.55±0.19^{NS}$	0.58±0.31
18	2-Oxoglutarate	$0.87±0.31^{NS}$	0.99±0.22
19	Oxypurinol	$0.17±0.04^{NS}$	0.19±0.06
20	Raffinose/Stachyose	$23.80±6.80^{NS}$	25.63±6.44
21	Succinate	$0.52±0.16^{NS}$	0.57±0.21
22	Sucrose	$36.17±3.35^{NS}$	36.83±4.74
23	Tartarate	0.79±0.19*	0.53±0.18
24	Tryptophan	1.97±0.28*	2.26±0.28
25	Valine	1.94±0.32*	1.54±0.44

Values in the table represent the total area normalization binning values, which means relative bin signal intensities of each compound were normalized by sum of the bin signal intensities and multiplying it by 1,000. Superscript characters (*) indicate significant differences between samples of the two groups, determined by Independent t-test; NS, not significant.

Table S5. Variable importance projection (VIP) values of metabolites for discriminating different origins of the Chinese soybean samples

No.	Compounds	VIP value
1	Sucrose	1.422
2	2-Hydroxyisobutyrate	1.237
3	Leucine	1.236
4	Malonate	1.214
5	Tartarate	1.194
6	Glucose	1.184
7	Succinate	1.156
8	Hypoxanthine	1.068
9	Acetate	1.049
10	Citrate	1.034
11	Choline	1.032
12	Isoleucine	0.998
13	Alanine	0.988
14	2-Oxoglutarate	0.969
15	Galactarate	0.960
16	Valine	0.913
17	Acetoacetate	0.905
18	Oxypurinol	0.848
19	Formate	0.812
20	Tryptophan	0.788
21	Asparagine	0.785
22	Aspartate	0.780
23	Fumarate	0.705
24	Glutamate	0.696
25	Raffinose/Stachyose	0.463

Table S6. Parameters of PLS-DA models based on various VIP cut-off values for discriminating different origins of the Chinese soybean samples based on standardized area normalization and UV scaling methods

VIP cut-off	Number of variables	Number of component	R^2Y	Q^2Y	R^2Y intercept	Q^2Y intercept
**Standardized area normalization, UV scaling**						
0	25	6	0.898	0.651	0.348	-0.821
>0.7	23	5	0.888	0.690	0.273	-0.661
>0.8	19	5	0.898	0.745	0.249	-0.658
>0.9	17	3	0.767	0.654	0.157	-0.351
**>1.0**	**11**	**4**	**0.887**	**0.789**	**0.151**	**-0.480**
>1.1	7	3	0.784	0.716	0.082	-0.344

Number of components obtained from autofit function in SIMCA software; VIP, variable importance projection; UV, unit variance; The bold characters indicate the selected optimal model parameters.

Table S7. Relative levels of metabolites in Chinese soybean samples.

No.	Compounds	Northeastern region	Middle region	Southern region
1	Acetate[2]	4.67±0.43*	7.39±1.54$^{\#}$	15.46±10.18$^{\#}$
2	Acetoacetate[2]	1.20±0.45^{NS}	1.11±0.27*	1.81±0.63$^{\#}$
3	Alanine[2]	1.22±0.45*	1.89±1.16^{NS}	2.89±1.50$^{\#}$
4	Asparagine[2]	2.85±0.93*	7.92±8.78^{NS}	10.31±5.19$^{\#}$
5	Aspartate[2]	6.59±2.33*	12.79±10.82^{NS}	15.06±5.64$^{\#}$
6	Choline[1]	378.20±35.88*	392.04±18.52*	340.67±40.91$^{\#}$
7	Citrate[2]	205.16±25.90^{NS}	199.17±15.61^{NS}	213.89±49.52^{NS}
8	Formate[1]	0.40±0.05^{NS}	0.41±0.08^{NS}	0.39±0.07^{NS}
9	Fumarate[1]	0.65±0.18*	0.59±0.07^{NS}	0.51±0.06$^{\#}$
10	Galactarate[1]	6.29±1.14*	6.66±1.02*	4.81±1.12$^{\#}$
11	Glucose[1]	3.18±0.82*	2.00±0.29$^{\#}$	2.03±0.64$^{\#}$
12	Glutamate[1]	30.66±5.56^{NS}	30.71±4.39^{NS}	27.29±2.57^{NS}
13	2-Hydroxyisobutyrate[1]	3.56±0.37*	4.07±0.72^{NS}	4.53±0.43$^{\#}$
14	Hypoxanthine[1]	2.86±0.66*	1.95±0.48$^{\#}$	1.52±0.51$^{\#}$
15	Isoleucine[2]	2.11±0.56^{NS}	1.70±0.30^{NS}	2.22±0.55^{NS}
16	Leucine[1]	21.86±1.89*	16.58±3.15$^{\#}$	15.29±2.57$^{\#}$
17	Malonate[2]	1.56±0.47*	2.01±0.81*	3.38±1.08$^{\#}$
18	2-Oxoglutarate[1]	5.96±0.50^{NS}	5.57±0.65^{NS}	6.87±2.12^{NS}
19	Oxypurinol[2]	0.90±0.25^{NS}	0.73±0.17^{NS}	0.69±0.21^{NS}
20	Raffinose/Stachyose[1]	115.28±25.38^{NS}	104.10±27.56^{NS}	100.04±29.88^{NS}
21	Succinate[2]	2.17±0.49^{NS}	1.99±0.34*	2.72±0.64$^{\#}$
22	Sucrose[2]	172.70±15.60*	178.39±8.68*	114.30±16.63$^{\#}$
23	Tartarate[1]	3.12±0.34*	2.18±0.60$^{\#}$	1.45±0.45^{+}
24	Tryptophan[1]	10.23±1.04*	9.37±1.42^{NS}	8.71±1.69$^{\#}$
25	Valine[1]	6.57±1.51^{NS}	5.57±1.08^{NS}	6.81±1.44^{NS}

Values in the table represent the standard area normalization binning values, which means relative bin signal intensities of each compound were normalized by dividing the bin signal intensities of internal standard (IS) and multiplying it by 100. Superscript characters (*, #, and +) indicate significant differences among three group samples, determined by the one way-ANOVA ($p < 0.05$, One way analysis of variance, marked by “^{1}”) and Kruskal-Wallis test ($p < 0.017$, marked by “^{2}”). NS; not significant.
